# Toland’s Dental Depot

**Published:** 1856-12

**Authors:** 


### TOLAND'S DENTAL DEPOT.

The veritable, universal correspondent for the whole profession, South and West, the genuine "J. M. Brown, per Jno. T. Toland," who has so often hoped that our goods would "reach us in good time and please," is the proprietor of "Toland's Dental Depot." His stock is extensive and well selected; and, besides, he is exclusive agent for Sherwood's instruments. His goods and prices we presume will be found, in all cases, satisfactory. See *outside* page of cover.

